# Supplementary material for: Multi-color photonic integrated circuits based on homogeneous integration of quantum cascade lasers
Source: Nat Commun. 2025 Apr 15;16:3563. doi: 10.1038/s41467-025-58905-0 (PMC12000614; doi:10.1038/s41467-025-58905-0)
Supplement: Supplementary file 1 — Supplementary Information [file 41467_2025_58905_MOESM1_ESM.pdf]

Supplementary Information

1. Influence of passive core thickness on threshold current densities of quantum cascade lasers fabricated from the photonic integrated circuit wafer

A deviation in the passive core thickness was observed during the fabrication of the two-color PICs. SEM images confirmed that the passive waveguide core layer was approximately 1330 nm thick, 6.5% above the target thickness of 1250 nm. While this issue can easily be controlled for in the future growths, the unexpected change in the passive waveguide core thickness led to the unpredicted increase in the threshold current densities J_{th} of the DFB QCLs processed from the PIC wafer used for our current work. As a result, only one of the two lasers on the PIC achieved CW operation at room temperature.

The threshold current density of a semiconductor laser is inversely proportional to the modal overlap Γ [36]:

$$J_{th} = \frac{\alpha_m + \alpha_w}{\Gamma g}. \quad (1S)$$

Here α_m is the mirror losses, α_w is the waveguide losses, and g the differential gain coefficient. Although the 5-mm-long, 10-µm-wide ridge-waveguide reference devices situated directly on top of the unetched passive waveguide layer (as shown in Fig. 4 of the main text) had low threshold current densities, the 7-8 µm wide DFB devices with sidewall corrugation integrated with the passive components exhibited increased threshold current densities due to mode leakage from the QCL core into the passive waveguide core and resultant decrease in the value of Γ. This prevented CW operation of the 7.3 µm DFB QCLs in the PIC at room temperature.

To investigate the effect of ridge width on the devices' performance, we characterized more reference devices of the length equal to that of the lasers reported in Fig. 4 of the main text (5 mm, to keep α_m constant) but with reduced ridge widths of 8 µm and 6 µm. Figure 1S (a,b) shows the light-current characteristics for the 5-mm-long Fabry-Perot ridge-waveguide lasers with the ridge widths of 10 µm, 8 µm, and 6 µm. The data is shown for devices processed from the 6.25 µm QCL sections of the PIC wafer and devices processed from the 7.3 µm QCL sections of the PIC wafer. A significant increase in threshold current density was observed for the devices with 8 µm ridge widths. Devices 6 µm ridge widths did not achieve lasing. These experimental findings are confirmed by COMSOL simulations of the

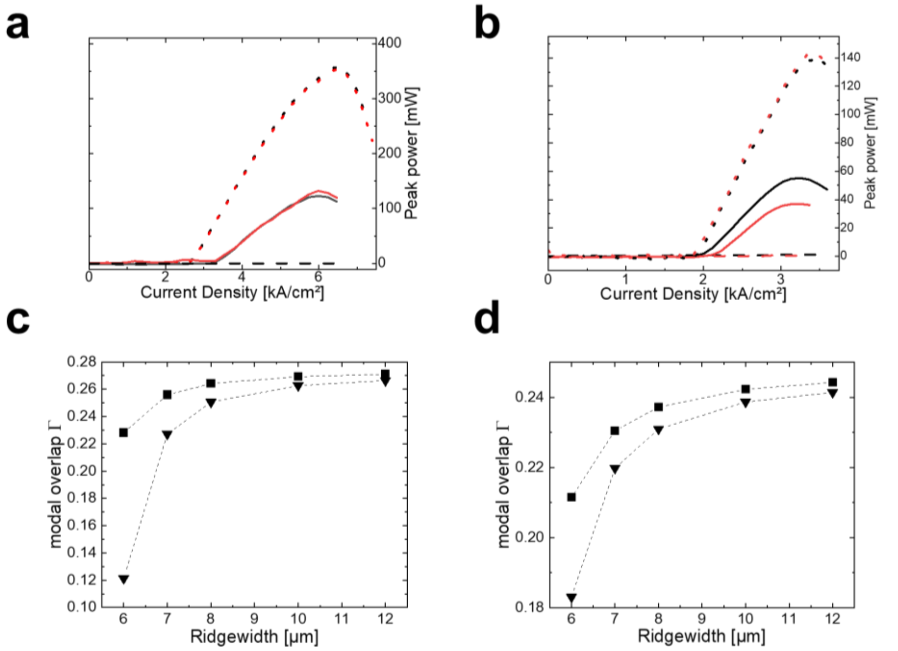

Supplementary Figure 1. Influence of the passive core thickness on threshold current densities of the photonic integrated circuit lasers. (a,b) Light-output-current characteristics of 5 mm long ridge-waveguide quantum cascade lasers with different ridge widths of 10 µm (dotted lines), 8 µm (solid lines), and 6 µm (dashed lines) processed from the two-color photonic integrated circuit wafer. Results for the 6.25 µm quantum cascade laser (a), and for the 7.3 µm quantum cascade laser (b) are shown. (c,d) Simulated overlap factors for the quantum cascade lasers of different ridge widths processed from the photonic integrated circuit wafer. The data is shown for the case of the passive waveguide core thickness being the target 1250 nm (squares) and the actual 1330 nm thickness (triangles). Panel (c) shows the results for the λ=6.25 µm quantum cascade lasers, panel (d) shows the results for the λ=7.3 µm quantum cascade lasers.

modal overlap of the fundamental TM_{00} mode with the QCL active region. The data is shown in Fig. 1S (c,d) as a function of devices' ridge widths for the case of both the target thickness and the 6.5% higher thickness of the passive waveguide core. The results indicate up to factor of two decrease in the laser mode overlap with the QCL active region. We thus expect that the PIC laser performance can improved considerably by maintaining the accurate control of the passive waveguide core thickness.

2. Suppression of power oscillations during continuous-wave operation by anti-reflection coating of the passive waveguide facet

Power output oscillations where observed for the PIC operating in CW regime in Fig. 7(d) of the main text. The oscillations were attributed to the etaloning effect due to light reflection from the output facet of the passive waveguide. A dielectric anti-reflection coating may be used to suppress waveguide facet reflectivity to avoid the etaloning effect. We have deposited anti-reflection coatings on the output waveguide facet of the PIC tested in Fig. 7(d) and indeed observed a complete removal of the etaloning effect in the light output-current characteristics. Unfortunately, CW operation of the PIC with anti-reflection coating could only be observed with some cooling as shown in Fig. 2S. As shown in Fig. 2S, the device has a linear slope efficiency over a range of 3.2 W in electrical power consumption, which would result in approximately 3.5 oscillations of the output power for an uncoated device (cf. Fig. 7(d)). Improving device waveguide configuration as discussed above and increasing the DFB grating coupling strength will enable laser operation at room temperature.

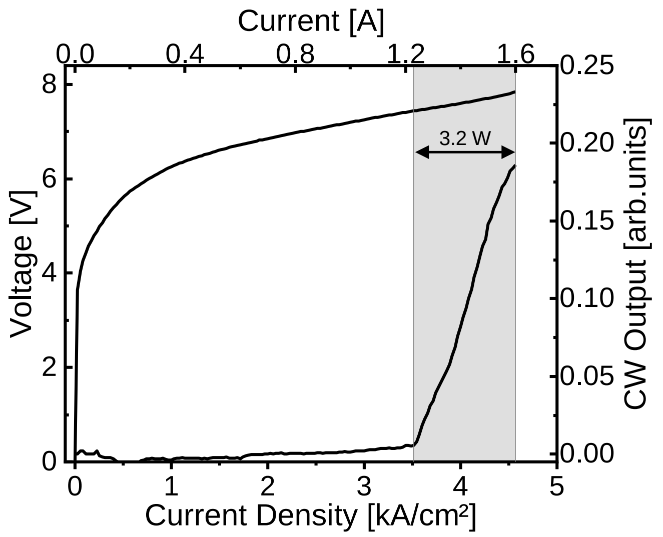

Supplementary Figure 2. Continuous-wave light-output-current characteristics of the photonic integrated circuit tested in Fig. 7 with anti-reflection coating deposited on the output facet. The anti-reflection-coated device shows a linear slope efficiency for the entire range of 3.2 W of power consumption highlighted by the light grey shaded region. Measurements are performed at 200°K.
